# Building recommendations for Mandatory Continuing Medical Education in developing countries: A nominal group study of regional experts

**DOI:** 10.12688/mep.21451.1

**Published:** 2026-01-05

**Authors:** Farhan Saeed Vakani, Kerry Uebel, Chinthaka Balasooriya, Apo Demirkol

**Affiliations:** 1 Dow Institute of Health Professionals Education, Dow University of Health Sciences, Karachi, Sindh, Pakistan; 2 School of Population Health, University of New South Wales, Sydney, New South Wales, Australia

**Keywords:** Mandatory Continuing Medical Education, developing countries, physicians, nominal group, re-registration, Life-long learning, South-east Asian Region, Eastern Mediterranean region

## Abstract

**Introduction:**

Internationally, mandatory Continuing Medical Education (CME), tied to re-registration, has enhanced physicians’ skills in developed nations. However, success rates have been inconsistent in developing countries. The current literature highlights the disparity in the availability of CME activities in the Southeast Asian and Eastern Mediterranean regions. This study aims to identify the most crucial elements that will serve as a blueprint for the optimal implementation of mandatory CME in developing countries.

**Methods:**

Following the development of 31 recommendations through a review of the literature of 33 countries in Southeast Asian and Eastern Mediterranean regions regarding the status of CME, a narrative review of five of these countries that have implemented mandatory CME, and in-depth exploration of the experiences and practices of CME providers in Pakistan, we used a nominal group technique with regional experts to identify those that are most crucial. An online meeting was held to establish recommendations for implementing CME in developing nations using a hypothetical developing country. Two rounds of discussion occurred during this consensus-building process.

**Results:**

The expert panel identified eight recommendations within the themes of educational design, delivery, and CME governance as the most relevant for implementing mandatory CME in developing nations. These include topics of national significance determined by a central body as desirable components of a CME programme, a combination of online and face-to-face methods, support for developing institutions that are currently active in providing CME in rural settings, accrediting a wide range of CME providers, forming a national CME committee under the national physician regulatory body, a predetermined phase-in period before full implementation of mandatory CME, developing guidelines to regulate and control commercial sponsorship of CME events, and broadening the definition of full disclosure.

**Discussion:**

These eight recommendations are likely to benefit developing nations that may be attempting to implement or revamp mandatory CME.

## Introduction

Our knowledge-driven society and ongoing developments in the medical field place a significant responsibility on physicians to regularly update and enhance their skills. Continuous Medical Education (CME) serves as a platform for effective learning and professional growth through activities that are designed to expand knowledge and improve skills. It also contributes significantly to social networking and relationships, which are crucial for physicians to provide better services to their patients and their profession ^1, 2^.

Several reports and reviews have outlined the effectiveness of CME in improving physicians’ performance ^3, 4^, and its impact is evident through mandatory continuing educational programs implemented in developed regions of the world ^5^. Some large developing countries, including India, Indonesia, and China, have relatively well-established systems in the form of mandatory CME but have faced various challenges in its implementation ^6^. Other large developing countries, such as Pakistan, have not been able to implement mandatory CME despite several attempts ^7– 9^.

This paper builds on a body of work conducted previously by the authors that included a scoping review of the status of CME in all 33 countries in Southeast Asian and Eastern Mediterranean regions ^10^, a narrative review of five of these countries that have implemented mandatory CME, and in-depth exploration of experiences and practices of CME providers in Pakistan ^11^. This research led to the development of 31 recommendations. This study was conducted in March 2021 during the COVID-19 pandemic, aiming to explore regional experts' views on the relevance of these 31 recommendations and to identify which are of the highest priority. Our goal is to guide the best practices for mandatory CME in developing nations.

## Materials and methods

### Nominal group technique

We used the modified nominal group technique and invited eight regional experts in medical education to identify the most relevant recommendations for implementing mandatory CME in developing countries. The nominal group is a well-established technique for measuring and developing consensus using highly structured face-to-face meetings with relevant experts in a focus group setting ^12, 13^. We used a hypothetical case study that illustrates a developing country attempting to implement CME on a national scale after several previous attempts.

### Context and data sources

The 31 recommendations ( developed through the work cited above) were collated into seven categories under three broad themes: educational design, educational delivery, and Governance of CME.

***A- Educational Design***

**I. Category - Mechanisms to identify priority topics for inclusion in CME**

1   Topics of national significance determined by a central body as desirable components of a CME programme

2   Wise-man approach (Needs described by the departmental heads, professors, teachers)

3   Learner-led education through self-reflection

4   Educational priorities determined by providers through a CPD ePortfolio

5   Educational priorities identified through Competency-based assessment test

**II. Category - Choosing appropriate CME Educational Formats**

1   Face-to-face conferences, seminars and, symposiums

2   Online conferences, seminars and symposiums (synchronous online)

3   Digital resources available online for use when needed by physicians (asynchronous online)

4   Workplace-based learning (networking and peer review of practice within the workplace)

5   Combination of online and face-to-face methods: online delivery of content supported by face-to-face discussions of complex issues

***B- Educational Delivery***

**III. Category - Ensuring access to CME for rural physicians**

1   Provide support to develop institutions which are currently active in providing CME in rural settings

2   Provide incentives to mobilise experienced physician CME teachers to rural practices

3   Provide online educational activities

4   Support the development of high-quality online educational activities, supplemented with relevant IT training

5   Provide incentives to rural physicians to attend urban training sessions (leave, pay, etc.)

**IV. Category - Ensuring equitable access to accredited CME activities**

1   Accrediting a wide range of CME providers

2   Accrediting a wide range of educational activities, e.g., workplace activities

3   Decentralising and devolving administrative responsibilities to larger providers

4   Incentivise regional collaboration between CME providers and health institutions to share experience

***C- CME Governance***

**V. Category - Establishing a national regulatory structure**

1   Appoint a group of reputed medical educators to establish a national CME committee

2   Form a national CME committee under the national physician regulatory body

3   Consult medical institutions with prior experience in providing CME to guide the process of establishing a national CME committee

4   Consult national physician associations to guide the process of establishing a national CME committee

5   Review and strengthen existing structures rather than create new structures

**VI. Category - Strategies to increase the success of the introduction of CME**

1   Predetermined phase-in period before full implementation of mandatory CME

2   Implement on a regional or state-by-state basis

3   Implement CME attendance as a link to promotion in institutions as first step

**VII. Category - Ensuring unbiased funding**

1   Develop guidelines to regulate and control commercial sponsorship of CME events

2   Broaden the definition of full disclosure (i.e., not only the physician speaker to disclose but CME providers to disclose the full amount and nature of support)

3   Train and empower physicians to critically analyse the funding bodies and make their own ethical decisions

4   Provide increased support from the government for CME to counter the influence of industry

### Participants

We invited eight regional experts in medical education from SEAR and EMR to explore their understanding of the appropriateness of the 31 draft recommendations and seek their views to identify the highest-priority items.

The experts were invited to participate by sending an invitation to members of the Asian Pacific Medical Education Network (APMENet), Pakistan Medical Commission (PMC), and The Qatar Council for Health Practitioners (QCHP). These organizations were approached as they represent experts in medical education within the SEAR and EMR regions. Written informed consent for participation in the research was obtained from participants who agreed to take part and were invited to a 90-minute online meeting.

### Process

The meeting was structured in two rounds (Round 1 & Round 2).

Round 1 included the following stages:

1. *Silent reflection:* In the first stage of round 1, participants were asked to read and reflect individually on a hypothetical case study. This hypothetical case described a developing country called “Bibakistan” that was trying to implement CME nationally after making several attempts. According to this hypothetical case, national authorities chose one recommendation from each of the seven categories to ensure successful implementation. The national body of the country requested regional experts' advice on the proposed recommendations. To construct this hypothetical case, the research team randomly chose one recommendation from each of seven categories. (see the hypothetical case in Appendix 1).
2. *Round Robin:* At this stage, each participant was asked to read through and consider, individually and without discussion, the advice they would give to this hypothetical country by looking at the 31 recommendations addressing the seven broad categories (there were a minimum of three and a maximum of five recommendations in each category). This process was designed to help participants become familiar with the 31 recommendations and consider their applicability to a developing-country context (by considering the hypothetical scenario). They were then asked, again without any discussion, to rank the recommendations in each category, from 1 being “most important” to 5 being “least important, ” to successfully implement CME in developing nations (see Table 1). The rankings from each participant were captured, and the cumulative results for each recommendation were illustrated through bar graphs.

**Table 1.** Nominal Group Results.

		Round 1 Average Scores (n=5)	Round 2 Average Scores (n=7)
**A-Educational Design**			
**Category I Mechanisms to identify priority topics for inclusion in CME**			
**1**	Topics of national significance determined by a central body	4.6	4.7
**2**	Wise-man approach	2.0	2.6
**3**	Self-reflection	3.0	3.0
**4**	CPD ePortfolio	3.2	2.9
**5**	Competency-based assessment test	2.2	1.9
**Category II Choosing appropriate CME Educational Formats**			
**1**	Face-to-face	1.8	2.4
**2**	Synchronous online	3.0	3.0
**3**	Asynchronous online	3.6	3.0
**4**	Workplace-based learning	3.4	2.3
**5**	Combination of online and face-to-face	3.2	4.3
**B- Educational Delivery**			
**Category III Ensuring access to CME for rural physicians**			
**1**	Institutions active in providing CME in rural settings	2.6	4.3
**2**	Mobilise experienced physician CME teachers to rural practices	2.6	3.3
**3**	Provide online educational activities	3.8	3.1
**4**	High-quality online educational activities, with relevant IT training	3.4	1.9
**5**	Incentives to rural physicians to attend urban training sessions	2.6	2.4
**Category IV Ensuring equitable access to accredited CME activities**			
**1**	Accrediting a wide range of CME providers	3.4	3.7
**2**	Accrediting a wide range of educational activities	3.4	2.9
**3**	Decentralising and devolving administrative responsibilities to larger providers	1.6	1.7
**4**	Incentivise regional collaboration between CME providers and health institutions	1.6	1.7
**C-CME Governance**			
**Category V Establishing a national regulatory structure**			
**1**	Appoint a group of reputed medical educators	3.2	2.9
**2**	A CME committee under the national physician regulatory body	4.0	3.9
**3**	Consult medical institutions with prior experience in providing CME	3.6	3.3
**4**	Consult national physician associations	2.4	2.9
**5**	Review and strengthen existing structures	1.8	2.1
**Category VI Strategies to increase the success of the introduction of CME**			
**1**	Predetermined phase-in period	2.8	2.6
**2**	Implement on a regional or state-by-state basis	1.6	2.0
**3**	Implement CME attendance as a link to promotion in institutions	1.6	1.4
**Category VII Ensuring unbiased funding**			
**1**	Develop commercial sponsorship guidelines	2.4	2.7
**2**	Broaden the definition of full disclosure	2.4	2.7
**3**	Train physicians to critically analyse the funding bodies	2.4	2.3
**4**	Support from the government for CME	2.8	2.3

3. *Clarification:* An expert in medical education from the research team (CB) then facilitated a discussion with all participants based on the results of the rankings. The participants were asked to clarify and discuss the reasoning behind their ranking of individual recommendations. Participants' conversations and comments were later transcribed by (FV) and validated by the research team.

Round 2 include the following two stages:

1. *Re-ranking of recommendations:* Following the discussion, participants were asked to re-rank the recommendations to capture any changes in views subsequent to the discussion.
2. *Focussed reflection:* re-ranking was shared with the participants and illustrated through graphs to show any potential changes in the results.

### Data analysis

The average score for each recommendation was calculated for the seven categories in both rounds 1 and 2. The following process was used to calculate the mean and identify the highest-ranked items within each category: where there were five recommendations in a category, five points were awarded for each participant who had ranked the recommendation first, four points for those who ranked it second, three points for ranking it third, etc. The scoring was adjusted for other categories: where there were four recommendations in a category, only four points were awarded for the first ranking, and where there were three recommendations, only three points were awarded for the first ranking. The scores for each recommendation were totalled and then divided by the number of participants to give an average ranking score. The results for rounds 1 and 2 were reported using the average scores. The minimum to maximum possible ranges for average scores were 1–5 where there were five recommendations in a category, 1–4 where there were four recommendations and 1–3 where there were three recommendations in a category.

## Results

Eight medical educators participated in the online nominal group process. They represented different organizations in the region, namely, APMENet (6), PMC (1), and QCHP (1). All participants actively participated in the discussion; however, due to technical connectivity issues, only five participants were able to complete their rankings in the first round. In the second round, seven participants completed the rankings. The findings are presented in the following seven core categories within three broad themes.

A. Educational Design
  I. Mechanisms to identify priority topics for inclusion in CME
  II. Choosing appropriate CME Educational Formats
B. Educational Delivery
  III. Ensuring access to CME for rural physicians
  IV. Ensuring equitable access to accredited CME activities
C. CME Governance
  V. Establishing a national regulatory structure
  VI. Strategies to increase the success of the introduction of CME
  VII. Ensuring unbiased funding

Table 1 presents the average scores for all 31 recommendations in the seven categories for rounds 1 and 2, enabling us to identify the most relevant strategies recommended by the panel to develop best practices for implementing mandatory CME in developing countries.

***A. Educational design***

**Category I. Mechanisms to identify priority topics for inclusion in CME**

The panel was presented with five recommendations under this category, and the maximum possible average score was 5. “Topics of national significance to be determined by the central body as desirable components of a CME program (Category I, rec. 1)” was ranked the highest among the series of recommendations in Category 1 during Round 1, with an average score of 4.6.

While discussing the recommendations and their fit for developing country contexts, the panel supported the need to have a national direction on topics to establish the CME programme. One medical educationalist commented,

*“I think from my experience as we're talking about a system, i.e., developing and facing some difficulties, positions as health care professionals in general if they're not living in a well-established CME system, they need guidance in the very beginning”.*

A member of the expert panel commented that the ‘Wiseman approach’ could be another important strategy among the given recommendations. This method for identifying learning needs involves consulting departmental heads, teachers, governing bodies, or scientific committees to gather valuable insights that help create personalized learning experiences for the target audience ^6^. However, participants also discussed that the Wiseman approach would bring up problems due to the lack of evidence-based research in CME and suggested that as the national CME programme evolves, other approaches that are more dependent on the individual physician identifying needs could be used. Another expert, while commenting about the purpose of CME and physicians learning needs highlighted the importance of needs assessment through CPD ePortfolios. He further explained that this depends on the country’s context. If it is a large country with different needs across different areas, then the topics needed would be difficult to determine at a national level and that portfolios would identify physicians learning needs. Another panel member responded as follows.

*“I'm not saying that the e-portfolio is not important, it is very important, provided that they understand how to develop the portfolio and they are competent enough to understand what to include and what is relevant to their needs, so they need guidance and education and provided that the CPD provider or the body that is planning utilize it in the right way.”*

 In round 2, “Topics of national significance to be determined by a central body as desirable components of a CME program (Category I, rec. 1)” remained the highest-ranked recommendation, with an average score of 4.7 among the series of recommendations in category 1. This item was selected for inclusion in the final recommendation.

**Category II. Choosing appropriate CME Educational Formats**

The panel considered five recommendations within this category, and the maximum possible average score was 5. In round 1, the recommendation was to include “digital resources available online for use when needed by physicians (Category II, rec. 3)” was ranked highest with an average score of 3.6.

During the clarification round, the panel discussed the advantages of online digital resources available asynchronously as the need of the hour because of the COVID pandemic and because physicians may not be able to use the real-time online platform or the places because of lack of IT skills or where Internet connectivity is a challenge. There was some discussion on the need for a combination of online and face-to-face presentations, as there are specific skills and competencies that can be better taught through face-to-face interactions.

A Professor in medical education commented,

*“I am a firm believer that the content is leading, and format should adapt to the content, so some content is best done on asynchronously online or synchronously online. And some content is best-done face to face.”*

A Professor in dental education added,

*“This is the future. The Covid-19 has taught us something that online is probably something we've to incorporate.”*

In round two, there was a clear consensus that a “combination of online and face-to-face educational formats (Category II, rec. 5)” would be the best recommendation with an average score of 4.3.

***B. Educational delivery***

**Category III. Ensuring access to CME for rural physicians**

Five recommendations were presented to the panel for this category, and the maximum possible average score was 5. In round 1, the recommendation to “Provide simple online educational activities (Category III, rec. 3)” Ensuring access to rural physicians was ranked highest, with an average score of 3.8.

During the discussions, the panel discussed most of the recommendations. They argued that other strategies were more resource-intensive and discussed issues of practicality. An expert commented on the advantage of having experienced physicians go to rural areas to teach, but others talked about the practical difficulties of such a plan.

*“it's important to bring experienced physicians to the rural hospitals at least in the beginning to ensure that they feel part of the national system, they're not left out, and of course if we start thinking cost-effective,……… bringing a small number of experienced physicians to the rural areas is much more cost-effective compared to providing incentives to the physicians to attend the sessions and can be considered as the training of the trainers' option as well.”*

Despite the initial high ranking of the recommendation to provide online activities for rural physicians, the members acknowledged that the lack of resources and Internet connectivity as well as lack of Internet skills were issues that would be a barrier to providing online educational activities in rural areas.

In Round 2 after this discussion, the highest ranked recommendation had changed with the clear consensus that “providing support to develop institutions which are currently active in providing CME in rural settings (Category III rec. 1)” would be the most relevant recommendation, with an average score of 4.3. It should be noted that this item was ranked third in the first ranking and moved to the highest ranking in Round 2.

**Category IV. Ensuring equitable access to accredited CME activities**

Four recommendations were considered by the panel for this category, and the maximum possible average score was 4. In Round 1, the two recommendations were highly ranked, with an average score of 3.4 each. They were “Accrediting a wide range of providers (Category IV rec. 1)” and “Accrediting a wide range of educational activities (Category IV rec. 2)”.

Both recommendations were discussed, with some members pointing out that implementing both options, that is, accrediting individual CME activities from smaller providers as well as accrediting larger CME providers, would result in the broadest range of choices. The recommendation to “Accredits a wide range of CME providers (Category IV rec. 1)” scored the highest, with an average score of 3.7 in Round 2.

***C. Governance***

**Category V. Establishing a national regulatory structure**

The panel considered five recommendations within this category, and the maximum possible average score was 5. In Round 1, the two recommendations had high scores:

- “Forming a national CME committee under the national physician regulatory body (average score of 4.0) Category V rec. 2”.
- “Consulting medical institutions with prior experience in CME to guide the process of establishing a national CME committee (average score of 3.6) Category V rec. 3”.

There was a significant discussion on both recommendations. The members broadly supported the formation of a national CME committee under physicians’ regulatory bodies. They argued that this recommendation would involve bodies other than just medical institutions in developing the national CME system. There was some discussion on first drawing the functional and operational specifications for a national regulatory structure involving both government and private academic institutions, and healthcare service providers.

An expert, while emphasising the importance, said,

*“I think we need to have something at a national level because this is really good in collecting information from different institutions and different bodies and the authorities. So we're not limiting ourselves to medical institutions, but we're also gathering data from other institutions.”*

In round two, the highest-ranked recommendation was to form a national CME committee under the national physician regulatory body (Category V rec. 2)” with an average score of 3.9.

**Category VI. Strategies to increase the success of the introduction of CME**

The panel considered three recommendations in this category, and the maximum possible average score was 3. In round 1, the recommendation to “Include a predetermined phase-in period before implementing mandatory CME (Category VI, rec. 1)” was ranked highest with an average score of 2.8.

During the clarification phase, some members were of the view that implementing CME on a regional or state-by-state basis would create more buy-in, which might be more appealing and much more recognizable by the learners. On the other hand, they believed that implementation in one region may not necessarily be a model for other regions because of the varying contexts, resources, and educational needs of physicians.

While supporting the predetermined phase-in period, one of the panelists stated:

*“I think once you have developed a culture, then you can make it mandatory, but to start off within the embryonic stage, we need to nudge people along.”*

In round 2, there was still agreement among the experts that “Include a predetermined phase-in period before implementing mandatory CME (Category VI, rec. 1)” was the most relevant recommendation with an average score of 2.6.

**Category VII. Ensuring unbiased funding**

In this category, the panel considered four recommendations, and the maximum possible average score was 4. In round 1, the recommendation to “Provide support from the government for CME to counter the influence of industry (Category VII, rec. 4)” scored the highest, with an average score of 2.8.

During the discussion, the panel discussed that despite an expressed preference for government financial support for CME training to counter the influence of industry, the reality is that governments, especially in resource-constrained settings, cannot rely on providing funds for CME because of competing demands for funding. There has also been much discussion on other recommendations. An expert commented on the importance of training physicians in analyzing funding status. She quoted,

*“they’re the ones who attend activities, evaluate the activities…for any commercial bias and conflict of interest available in the activities. So, I believe that it's important to train physicians. They are the target audience; they are the ones who give the authorities and the educators, of what really happened during the activities.”*

Another member from the panel had some reservation and said,

*“I think it’s unfair to put physicians in this kind of situation where they are analysing the funding and all that. I think that should be determined either by a regulatory body or an institution rather than putting individual physicians in difficult positions*. *I would put full disclosure on top of training physicians. Because I think once the CME provider has a full disclosure, then the audience can make up their own mind or as to where the CME provider is coming from.”*

As a result, in round 2, two recommendations, Broaden, the definition of full disclosure (Category VII rec. 2)” and “Develop guidelines to regulate and control commercial sponsorship of CME events (Category VII rec. 1) gained equal support, with an average score of 2.7 each. As a result of this tie in round two, both items were included in the final recommendations.

## Discussion

The panel of experts discussed 31 possible recommendations in the seven areas identified as important issues in implementing CME in developing countries. After discussion, the experts reached a consensus on the eight most relevant recommendations. These recommendations are presented below under the three original conceptual themes.

A. Educational Design
  a. Topics of national significance determined by a central body as desirable components of a CME program (Category I rec. 1)
  b. A combination of online and face-to-face methods (Category II rec. 5)
B. Educational Delivery
  a. Provide support to develop institutions that are currently active in providing CME in rural settings. (Category III rec. 1)”
  b. Accrediting a wide range of CME providers (Category IV rec. 1)
C. CME Governance
  a. Form a national CME committee under the national physician regulatory body (Category V, rec. 2)
  b. A predetermined phase-in period before full implementation of mandatory CME (Category VI rec. 1)”
  c. Develop guidelines to regulate and control commercial sponsorship of CME events (Category VII rec. 1)
  d. Broaden the definition of full disclosure (i.e., not only the physician speaker to disclose but also CME providers to disclose the full amount and nature of support) (Category VII rec. 2)

***A. Educational Design***

The findings showed that the expert panel had a broad agreement on the recommendation for a central body to determine topics of national significance as desirable components of a CME program. This was supported by the rationale that in countries where there are no established CME systems, physicians usually face difficulties in identifying their educational needs for which they need directions, and would benefit from initial support on topics of national interest guided by the national body. The panel discussed how a country’s culture and context play a crucial role in defining the needs of learners. India is an illustrative example of a lower-middle-income country in the SEAR region that promotes national interest and national health programs through the State medical councils ^14^. These initiatives by the State Medical Council of India are an attempt to direct the scope of CME that is nationally valuable to ensure that doctors are regularly updated about important national health agendas and professional practice.

Under the category of appropriate educational formats, the recommendation for a combination of asynchronous online and face-to-face presentations gained the majority of support. The panel recognized the issues and problems associated with synchronous online activities and the convenience of attending asynchronous digital activities at their own time and pace. They felt that the format must adapt to the content because there are situations where some content can be best delivered online and, in some instances, face-to-face sessions are required. Therefore, it needs to be tailored to suit the infrastructure and the needs and availability of resources within the country. In fact, online activities that are overly dependent on technology pose challenges in connecting learners and instructors compared to traditional face-to-face education. Online education is often influenced by the educational environment and factors such as available support systems and learners’ prior experience with online learning. The logistical challenges faced by rural physicians deserve significant attention ^15^. Experts supported a blended approach that integrated relevant ICT tools with traditional face-to-face teaching. A developing country like India has successfully implemented a blended model, a combination of traditional methods and online activities for training rural surgeons, where long distances and few specialists cause particular challenges ^15, 16^. They proposed a training package that integrates various pedagogical approaches such as self-study and lecture sessions, observing operative procedures through live transmissions (synchronous) or recorded videos (asynchronous), and attending hands-on-experience sessions in local hospitals under supervision ^16^.

***B. Educational Delivery***

There was support for recommending online training as the most relevant strategy in the earlier round; there was a discussion of the challenges in relying on online training to deliver CME to rural physicians, particularly synchronous sessions. The challenges may include rural physicians' limited access to communication capability (such as broadband internet, cellular phone networks), unreliable and patchy internet connections, cost, maintenance, physicians and instructors' training, and support to remote sites ^15^. After robust deliberation of the challenges and practical issues associated with synchronous sessions and other proposed strategies, the panel reached agreement that “developing institutions active in providing CME to rural physicians” as the most relevant recommendation. It appears that other recommendations were more resource-intensive and may be less successful in reaching physicians practising in rural areas.

Under the category of ensuring equitable access to accredited CME activities, the panel considered the recommendation to accredit a wide range of CME providers relevant. It has been observed that countries that successfully implemented mandatory CME were usually supported by a wide range of local CME providers ^10^. The recommendation to accredit a variety of activities was discussed, as some organizations may lack the resources to become accredited providers, but still offer valuable educational activities. In Qatar, the QCHP accredits both CPD providers and individual CPD programs ^17^.

***C. CME Governance***

Experts found that the most relevant recommendation was to form a national CME committee under the national physician regulatory body. The panel shared their experiences that this recommendation would allow other national bodies to be part of this committee and share their data and experiences in developing the national CME system. The panel discussed that this committee should have a defined scope with functional and operational specifications and should involve members from government and private academic institutions, and healthcare service providers. Therefore, to facilitate dialogue and establish a strong foundation for the entire process, the establishment of a national CME body similar to that proposed in Canada is strongly recommended ^18^.

To increase the success of the introduction of CME, the introduction of the predetermined phase-in period before implementing mandatory CME was recommended as the most relevant by the panel. The consensus from the panel was that the predetermined phase-in-period would be a good way to develop a culture of CME and to sensitize and create buy-in from physicians. The panel identified several limitations with the recommendations to implement CME on a regional or state-by-state basis and using CME attendance for promotion in institutions. They indicated diverse resources and varying learning needs of physicians among different regions of the country and the absence of connections between practitioners and academic institutions as their main limitations. Indonesia in SEAR countries and Kuwait and Qatar in the EMR region are successful models in implementing mandatory CME nationally that underwent a predetermined structured phase-in period before full implementation of mandatory CME ^17, 19, 20^. India, on the other hand, progressed through regional implementation on a state-by-state basis due to delays in national legislation, lack of support from professional associations, and resistance to change by physicians ^21^. In a developing country context, where national decision-making may be delayed, regional implementation may be another viable option.

The two relevant recommendations to limit commercial bias are the development of national guidelines to regulate and control commercial sponsorship and the mandating of full disclosure of commercial interests by CME providers. The experts believed that asking and relying upon physicians to determine the funding status would not be effective, nor would a fair burden to place on physicians for which continuous training for critical appraisal and ethical decision-making is required ^18^. Instead, providers should provide full disclosure, which can help learners identify funding bias in the CME program. To ensure impartial funding, all providers and collaborators must indicate the support in total amount or in-kind contribution so that physician learners can analyze the degree of support and influence of the industry on CME programming ^18^. The development of guidelines may strengthen the system and aid in managing and regulating commercial sponsorship. Kuwait and Qatar are two important countries in the EMR region that have established clear guidelines for collaborating with commercial companies involved in sponsoring CME activities ^17, 19, 22, 23^.

## Conclusion

These eight recommendations are imperative for developing nations in their efforts to successfully implement mandatory continuing medical education.

## Institutional ethics approval

This study was approved by the University of New South Wales HREAP G: Health, Medical, Community, and Social (Ref No: HC180960).

## Figures and Tables

**Table:** 

Activity/Topic	Time in minutes
**Introduction of the process to the participants and welcome introductions**	15 minutes
**Round 1- Questionnaire first attempt**	15 minutes
**Discussion on (Each Question)**	5 – 6 minutes each Question (50 minutes max)
**Round 2- Questionnaire second attempt and sharing of results**	10 minutes

## Data Availability

Figshare: Discharge summary dataset, https://doi.org/10.6084/m9.figshare.30939434 ^24^ This project contains the following underlying data: Results Sheet ^24^ Data Report Round 1 Data Report Round 2 Related Materials ^24^ Round 1 Form Round 2 Form Data are available under the terms of the Creative Commons Attribution 4.0 International license (CC-BY 4.0)

## Appendix 1

**Hypothetical Case:** Country Bibakistan is a lower middle-income country in the South-East Asian region. They had previously made several attempts to introduce CME, but these efforts have had limited success. Following an extensive local consultation there is a proposed program for practicing physicians. Now they want to review this program with regional experts. The key features of the program are:

1. Priority topics for inclusion in CME programs were determined by a national body
2. Workplace-based learning (networking and peer review of practice within the workplace) was encouraged as one of the key approaches to delivering CME activities
3. A national regulatory structure was established by appointing medical educators with expertise and experience in CME
4. Access to CME for rural physicians was supported by mobilising experienced physician CME teachers to rural practices
5. Ethical and legal issues around CME funding were addressed by physicians to critically analyse the funding opportunities and make ethical decisions
6. To ensure the success of the introduction of the CME program, CME attendance was linked to promotion in institutions
7. Issues around equity of access were addressed by accrediting a wide range of educational activities including workplace-based activities as noted above.

This is a proposal by their national experts. The seven elements above are being reviewed within seven categories, which include other options that have been identified. Following the review, a revised program will be confirmed (it is possible that some elements of the current program will be retained, while others will be replaced).

## Appendix 2: Instructions to the nominal group

“The purpose of this meeting is to seek your views on best practice to improve CME in developing nations.

We will be using the modified nominal group technique. This modified nominal group technique will be applied to the 31 key recommendations within the seven categories that were identified through the first stage of the research. This included a detailed review of the literature, scoping review, in-depth analysis of the significant countries implementing CME, and findings from a case study in Pakistan.

i.   The modified nominal group discussion will be conducted as a single online meeting of 90 minutes duration and will be structured as follows. Two rounds will be conducted.

ii.   In Round 1, a hypothetical CME program will be presented to the participants. Participants will be provided with several minutes to reflect on the hypothetical program.

iii.   Recommendations from each of the seven categories will be presented in the form of an online questionnaire. Each participant will be asked to rank their recommendations within each category. The aim of this step is to identify what experts consider most to least relevant from among the recommendations presented in each category.

iv.   Your ranking of recommendations within each category will be visually presented to the entire group. This data will be collated, and no identifying information will be visible or shared outside the research group. This step will help the group understand which items in each category are ranked most highly by the group.

v.   In (Round 2), a group discussion will be facilitated to discuss any variation in rankings and elicit reasoning behind the rankings by participants. Experts are encouraged to share diverse views and justifications. There are opportunities to exchange ideas and explore the reasons expressed by others.

vi.   Following this discussion, participants will be invited to re-rank the recommendations using a format similar to that used in Round 1. This is an opportunity for experts to change their views following the discussion. Please feel free to rank as you feel fit, based on your current views. While the aim is to reach a consensus within the group, you are encouraged to express your own views based on your experience, expertise, and what you have heard during the discussion.

vii.   The outcomes of the second round of ranking will be shared with the group.

viii.   During this discussion, the supervisory team volunteered to fulfill these responsibilities. (FV) would be leading, (CB) would be facilitating the discussion, (KU) will be taking the notes, and (AD) will critically manage the time.”

ix.   Time allocation
