# Supplementary material for: Building recommendations for Mandatory Continuing Medical Education in developing countries: A nominal group study of regional experts
Source: MedEdPublish (2016). 2026 Jan 5;16:2. [Version 1] doi: 10.12688/mep.21451.1 (PMC12902668; doi:10.12688/mep.21451.1)
Supplement: Peer review history

# Reviewer report: Reviewer response for version 1

**DOI:** 10.21956/mep.22977.r45275

**Published:** 2026-02-28

**Authors:** Jen-Hung Yang, Ming-Hao Liang

**Affiliations:** 1 Center for Medical Humanities, and School of Medicine, Chung Shan Medical University, Taichung, Taichung City, Taiwan; 2 Center for Medical Humanities, Chung Shan Medical University (Ringgold ID: 34899), Taichung, Taichung City, Taiwan

The overall logic of the study design is clear, and the attempt to reduce political sensitivity by using a virtual/fictitious case is a creative and pragmatic approach. However, I recommend further revision in the following areas: 

1. The study frames its scope as “developing countries,” and both the case selection and the sources of experts are primarily drawn from SEAR and EMR countries. Inferring findings from SEAR and EMR alone to “developing countries” broadly risks overgeneralization. It is recommended to explicitly clarify the intended scope of inference by clearly defining the operational definition of “developing countries” and the geographic and institutional contexts covered, to prevent overextension of the findings to countries or regions without comparable contexts in the *Introduction.*
2. Please provide the inclusion criteria for the Nominal Group and describe the academic backgrounds of the experts (e.g., clinicians, academic researchers, policymakers, etc.) so that readers can assess the representativeness of the perspectives.
3. In the *Participants* section, please elaborate that the sampling from SEAR and EMR is sufficient to reflect the diverse contexts of “developing countries”. Advise to explain the extent to which these experts’ perspectives reflect the institutional backgrounds and practical needs of other developing regions (e.g., Africa), and to provide limitations for inference.
4. In the *Process* section, although Appendix 1 briefly describes the background, the information provided is insufficient for readers to judge whether the case can reasonably reflect the diversity of developing-country contexts. Given the substantial differences among developing countries in politics, economy, governance structures, and resource conditions. The authors need to delineate the case’s context, design rationale, and key assumptions to support appropriate inferences.
5. In the *Round Robin* subsection, recommendations are ranked from 1 (most important) to 5 (least important) to assess their importance for promoting CME in developing countries. However,  inconsistent with the scoring method described in *Data analysis* (for example, when a category has five indicators, the first-ranked item receives 5 points and the fifth receives 1 point; when a category has four indicators, the first-ranked item receives 4 points and the fourth-ranked item receives 1 point) The authors should clarify to avoid confusion about the process and results interpretations.
6. Only five of eight experts completed the first-round scoring. Why did three participants not complete Phase 1, and elaborate on the effect on their dropout? In addition, authors should clarify whether experts who did not fully participate in Phase 1 were appropriate to include in the Phase 2 processes.
7. The study was conducted in March 2021, and the manuscript was written in 2026, resulting in a five-year gap. Over these five years, especially in the post-pandemic period, online technologies and user acceptance have changed substantially, which makes it difficult to interpret the finding of low acceptance of online courses in rural areas. Please discuss the time lag that may affect the results and interpretations related to advances in online technologies.
8. The strength of expert consensus differs markedly across categories in the Results. For example, in Category I, “Topics of national significance determined by a central body,” the mean score is 4.7 (max 5), indicating high consensus; in contrast, the mean scores for Category VII indicators range from 2.3 to 2.7 (max 4), suggesting more dispersed opinions. In addition to reporting means, we suggest the authors provide standard deviations or other dispersion metrics to help assess the consistency of consensus for each recommendation and to improve transparency in result interpretation.

Have any limitations of the research been acknowledged?

Partly

Is the work clearly and accurately presented and does it cite the current literature?

Partly

If applicable, is the statistical analysis and its interpretation appropriate?

Partly

Are all the source data underlying the results available to ensure full reproducibility?

Partly

Is the study design appropriate and is the work technically sound?

Partly

Are the conclusions drawn adequately supported by the results?

Partly

Are sufficient details of methods and analysis provided to allow replication by others?

Partly

Reviewer Expertise:

Professionalism, Curriculum, Accreditation, and AI Governance and Applications

We confirm that we have read this submission and believe that we have an appropriate level of expertise to confirm that it is of an acceptable scientific standard, however we have significant reservations, as outlined above.

# Reviewer report: Reviewer response for version 1

**DOI:** 10.21956/mep.22977.r45267

**Published:** 2026-02-24

**Authors:** Marcelo Garcia Dieguez

**Affiliations:** 1 Universidad Nacional del Sur, Bahía Blanca, Argentina

The topic is relevant, and the consensus approach is appropriate for policy questions where empirical evidence is heterogeneous. A major strength is the clear, practical output: a prioritised set of recommendations that can inform regulators and CME providers. The manuscript also demonstrates transparency. The scenario, forms, ethical approval, and the data availability statement pointing to an external repository are clearly presented.

 There are, however, several issues that raise concerns. Some may be addressable, while others may not; in that case, a dedicated limitations paragraph would be helpful.

 First, panel size and missingness are important. Only five participants completed Round 1 rankings due to connectivity issues, and seven completed Round 2. This threatens the robustness of the prioritisation. If there is a justification for this, please include it. The authors should also state how missing rankings were handled and add a simple sensitivity check showing whether the top-ranked items remain stable when analyses are restricted to Round 2 completers.

 Second, additional clarification on expert selection criteria and panel description would strengthen the manuscript. Please define the eligibility criteria for “expert,” report the size of the invited pool, and provide an anonymised panel table (country/region, role, and years of CME/CPD expertise).

 Third, the decision rule used to select the final eight recommendations should be made more explicit. Please clarify the selection threshold (e.g., top-1/top-2 per domain, a margin rule, or a score-based cut-off) and report score spreads for near-miss items to demonstrate the strength of preferences.

 As noted above, a dedicated limitations paragraph should be added (small panel, potential selection bias, connectivity constraints, and limits to generalisability). The conclusions should be calibrated to “recommendations identified by this panel,” avoiding overly imperative language.

 Finally, as a minor point, please verify that all grey literature references include stable URLs and access dates.

Have any limitations of the research been acknowledged?

Partly

Is the work clearly and accurately presented and does it cite the current literature?

Yes

If applicable, is the statistical analysis and its interpretation appropriate?

Not applicable

Are all the source data underlying the results available to ensure full reproducibility?

Yes

Is the study design appropriate and is the work technically sound?

Partly

Are the conclusions drawn adequately supported by the results?

Yes

Are sufficient details of methods and analysis provided to allow replication by others?

Yes

Reviewer Expertise:

Helth Human Resources, Competency based education. Residency Training, Assesssment

I confirm that I have read this submission and believe that I have an appropriate level of expertise to confirm that it is of an acceptable scientific standard, however I have significant reservations, as outlined above.

# Reviewer report: Reviewer response for version 1

**DOI:** 10.21956/mep.22977.r45268

**Published:** 2026-02-12

**Authors:** Saima Batool

**Affiliations:** 1 University of Lahore, Punjab, Pakistan

This manuscript addresses an important, relatively less researched issue. By adding the developing countries' data, building on the author's previous regional work increased its academic rigor.

 That said, there are several areas that need strengthening, like a small number of participants, which makes it necessary to justify the adequacy for consensus building.

 The author's need to explain the rationale for selecting only the highest-ranked recommendation per category rather than using a defined cut-off or including closely ranked items.

 The discussion occasionally moves from reporting consensus findings to making broader claims (e.g., that these recommendations are imperative).

Have any limitations of the research been acknowledged?

Partly

Is the work clearly and accurately presented and does it cite the current literature?

Yes

If applicable, is the statistical analysis and its interpretation appropriate?

Partly

Are all the source data underlying the results available to ensure full reproducibility?

Partly

Is the study design appropriate and is the work technically sound?

Yes

Are the conclusions drawn adequately supported by the results?

Yes

Are sufficient details of methods and analysis provided to allow replication by others?

Partly

Reviewer Expertise:

Health professions education, Faculty development, CME, Technology enhanced learning

I confirm that I have read this submission and believe that I have an appropriate level of expertise to confirm that it is of an acceptable scientific standard.

# Reviewer report: Reviewer response for version 1

**DOI:** 10.21956/mep.22977.r45271

**Published:** 2026-02-09

**Authors:** Barbara Jennings

**Affiliations:** 1 Retired from Medical School in 2025, University of East Anglia (Ringgold ID: 6106), Norwich, England, UK

**Peer Review: “** **Building recommendations for Mandatory Continuing Medical Education in developing countries: A nominal group study of regional experts”**

 The reviewer, Barbara Jennings, has no conflicts of interest to declare. She has expertise in faculty development, systematic review, and methodologies for consensus seeking in clinical education.

*Scope, Ethics, and Presentation*

 This article fits within the field of medical education and addresses the organization of continuous medical education (CME) and the principles of introducing mandatory CME programmes, with a focus on Southeast Asian and Eastern Mediterranean regions.  Institutional review board approval, data availability and author contributions are transparent; and the manuscript is very clearly written and presented.

*Contribution*

 This study builds on the findings from the research team’s recent systematic review which they cite in the introduction

 (Vakani FS, Uebel K, Balasooriya C, et al. The status quo of continuing medical education in South-East Asia and Eastern Mediterranean regions: a scoping review of 33 countries. J Contin Educ Health Prof. 2024; 44(1): 44–52.)

 They used a nominal group consensus method to generate a priority list for implementing and delivering CME programmes in developing countries. They recruited an expert group for a two round protocol which most participants completed – the output was a list of eight recommendations proposed for developing nations to follow for successful implementation mandatory CME. The recommendations span design, delivery and the ethical and practical governance of mandatory CME.

*Introduction: *this presents a clear rationale for the study with evidence cited from a range of useful studies and reports about CME organization in many diverse clinical settings. Theory-practice links are explored as well as the benefits of CME to clinical practice.

*Methods & Results*

 The nominal group methodology is described clearly and could be replicated by others given the detail and the presentation of documentation in the body of the article or in its appendices.

 Appendix 1 has the resources for the case-based discussion (this was prompted by trigger material with a hypothetical scenario and the recommendations published in the co-authors’ previous systematic review).

 Because online meetings were used, the researchers will have circumvented some of the usual organizational problems for face-to-face consensus methods involving experts recruited from different countries.

*Queries for authors: *

1. how many participants were in the invited expert pool; what were the demographic characteristics of the eight participants, e.g. with respect to age, sex and career stage?
2. Could the timing of the study affect the results, over and above the advantages recorded about online education and asynchronous activities noted in 2021 during the pandemic?

*Discussion and Conclusion*

 I found the overall findings and discussion very interesting and thought provoking. The challenges of defining specific governance arrangements were explored in detail and half of the final recommendations fell under the CME governance category (the remainder relating to educational design and delivery that scoring showed strong consensus for). The importance of blended teaching and learning strategies is highlighted as was the need for a national CME committee akin to a Canadian model, with the caveat that regional rather than nationwide rollout of CME may be necessary in developing countries.

*References*

 Useful citations as noted above, but a few minor revisions are needed e.g., to correct formats or add document hyperlinks e.g., see references 8, 9, 19 & 20.

*Overall Recommendation*

 Minor revisions advised.

 I suggest drafting a second version of the manuscript addressing the queries above, perhaps in a limitations section of the discussion or by adding more detail to the methods.

Have any limitations of the research been acknowledged?

No

Is the work clearly and accurately presented and does it cite the current literature?

Yes

If applicable, is the statistical analysis and its interpretation appropriate?

Not applicable

Are all the source data underlying the results available to ensure full reproducibility?

Yes

Is the study design appropriate and is the work technically sound?

Yes

Are the conclusions drawn adequately supported by the results?

Yes

Are sufficient details of methods and analysis provided to allow replication by others?

Yes

Reviewer Expertise:

expertise in faculty development, systematic review, and methodologies for consensus seeking in clinical education.

I confirm that I have read this submission and believe that I have an appropriate level of expertise to confirm that it is of an acceptable scientific standard, however I have significant reservations, as outlined above.

# Reviewer report: Reviewer response for version 1

**DOI:** 10.21956/mep.22977.r45269

**Published:** 2026-02-06

**Authors:** Devi Prasad Mohapatra

**Affiliations:** 1 Jawaharlal Institute of Postgraduate Medical Education & Research (JIPMER), Puducherry, India

Continuing medical education (CME) plays a vital role in enhancing physician performance by ensuring they remain updated with evolving medical knowledge, evidence-based practices, and technological advancements. CME helps bridge the gap between research and clinical practice, promoting lifelong learning and improved patient outcomes.

 In developing nations, implementing structured CME requirements poses challenges such as limited resources, lack of standardized accreditation systems, and variable access to learning platforms. However, with increasing digital access and global collaborations, there has been a  growing momentum to institutionalize CME programs, making them mandatory for re-licensure and promoting accountability in healthcare delivery.

 This present study works on the previous body of work (1,2) by the authors on similar themes where they have analysed the implementation of CME in developing countries and taking that the recommendations from previous studies further. The authors  have attempted to Identify issues from an expert panel through online discussions and categorise them under the themes of educational design, delivery, and cme  governance. The study methodology seems appropriate, and the recommendations given by this expert panel seems suitable for implementation.

*Further details on the expert panel members particularly their experience and field of interest in medical education as well as academic profile would aid  other researchers in replicating and taking this study forward.*

 1. Vakani FS, Uebel K, Balasooriya C, et al.: The status quo of continuing medical education in South-East Asia and Eastern Mediterranean regions: a

 scoping review of 33 countries. J Contin Educ Health Prof. 2024; 44(1): 44–52.

 PubMed Abstract | Publisher Full Text

 2. Vakani FS, Demirkol A, Uebel K, et al.: CME providers’ experiences and

 practices in Pakistan: a case study. BMC Med Educ. 2024; 2

Have any limitations of the research been acknowledged?

No

Is the work clearly and accurately presented and does it cite the current literature?

Yes

If applicable, is the statistical analysis and its interpretation appropriate?

Yes

Are all the source data underlying the results available to ensure full reproducibility?

Yes

Is the study design appropriate and is the work technically sound?

Yes

Are the conclusions drawn adequately supported by the results?

Yes

Are sufficient details of methods and analysis provided to allow replication by others?

Yes

Reviewer Expertise:

medical education, artificial intelligence in surgical training, aesthetic surgery, wound care, technology-enhanced learning, simulation-based education, craniofacial surgery, and clinical outcomes research

I confirm that I have read this submission and believe that I have an appropriate level of expertise to confirm that it is of an acceptable scientific standard.
